# Amendment of the Japanese consensus guidelines for autoimmune pancreatitis, 2020

**DOI:** 10.1007/s00535-022-01857-9

**Published:** 2022-02-22

**Authors:** Kazuichi Okazaki, Shigeyuki Kawa, Terumi Kamisawa, Tsukasa Ikeura, Takao Itoi, Tetsuhide Ito, Kazuo Inui, Atsushi Irisawa, Kazushige Uchida, Hirotaka Ohara, Kensuke Kubota, Yuzo Kodama, Kyoko Shimizu, Ryosuke Tonozuka, Takahiro Nakazawa, Takayoshi Nishino, Kenji Notohara, Yasunari Fujinaga, Atsushi Masamune, Hiroshi Yamamoto, Takayuki Watanabe, Toshimasa Nishiyama, Mitsuhiro Kawano, Keiko Shiratori, Tooru Shimosegawa, Yoshifumi Takeyama, Members of the Research Committee for IgG4-related Disease supported by the Ministry of Health, Labour, Welfare of Japan, Japan Pancreas Society

**Affiliations:** 1 Department of Internal Medicine, Kansai Medical University Kori Hospital, 8-45 Korihondori, Neyagawa, Osaka 572-8551 Japan; 2 Department of Internal Medicine, Matsumoto Dental University, Shiojiri, Japan; 3 Department of Internal Medicine, Tokyo Metropolitan Komagome Hospital, Tokyo, Japan; 4 Department of Gastroenterology and Hepatology, Kansai Medical University, Hirakata, Osaka Japan; 5 Department of Gastroenterology and Hepatology, Tokyo Medical University, Tokyo, Japan; 6 Neuroendocrine Tumor Centre, Fukuoka Sanno Hospital, International University of Health and Welfare, Fukuoka, Japan; 7 Department of Gastroenterology, Yamashita Hospital, Ichinomiya, Japan; 8 Department of Gastroenterology, Dokkyo Medical University School of Medicine, Tochigi, Japan; 9 Department of Gastroenterology and Hepatology, Kochi Medical School, Kochi University, Nankoku, Japan; 10 Department of Community-Based Medical Education, Nagoya City University Graduate School of Medical Sciences, Nagoya, Japan; 11 Division of Gastroenterology and Hepatology, Yokohama City University School of Medicine Graduate School of Medicine, Kanagawa, Japan; 12 Division of Gastroenterology, Department of Internal Medicine, Graduate School of Medicine, Kobe University, Kobe, Japan; 13 Department of Gastroenterology, Tokyo Women’s Medical University, Tokyo, Japan; 14 Department of Gastroenterology, Nagoya City University Graduate School of Medical Sciences, Nagoya, Japan; 15 Department of Gastroenterology, Tokyo Womens’ Medical University Yachiyo Medical Center, Yachiyo, Japan; 16 Department of Anatomic Pathology, Kurashiki Central Hospital, Kurashiki City, Okayama Japan; 17 Department of Radiology, Shinshu University School of Medicine, Matsumoto, Japan; 18 Division of Gastroenterology, Tohoku University Graduate School of Medicine, Sendai, Japan; 19 First Department of Internal Medicine, Shinshu University School of Medicine, Matsumoto, Japan; 20 Department of Gastroenterology, Shinshu University School of Medicine, Shiojiri, Japan; 21 Department of Public Health and Hygiene, Kansai Medical University, Osaka, Japan; 22 Department of Rheumatology, Kanazawa University Hospital, Kanazawa, Japan; 23 Tohto Clinic, Tokyo, Japan; 24 Department of Gastroenterology, South Miyagi Medical Center, Ohgawara, Japan; 25 Faculty of Medicine, Department of Surgery, Kindai University, Osakasayama, Osaka Japan

**Keywords:** Autoimmune pancreatitis, Guidelines, Diagnosis, Treatment, Delphi method

## Abstract

In response to the latest knowledge and the amendment of the Japanese diagnostic criteria for autoimmune pancreatitis (AIP) in 2018, the Japanese consensus guidelines for managing AIP in 2013 were required to be revised. Three committees [the professional committee for developing clinical questions (CQs) and statements by Japanese specialists; the expert panelist committee for rating statements by the modified Delphi method; and the evaluating committee of moderators] were organized. Twenty specialists in AIP extracted the specific clinical statements from a total of 5218 articles (1963–2019) from a search in PubMed and the Cochrane Library. The professional committee made 14, 9, 5, and 11 CQs and statements for the current concept and diagnosis, extra-pancreatic lesions, differential diagnosis, and treatment, respectively. The expert panelists regarded the statements as valid after a two-round modified Delphi approach with individually rating these clinical statements, in which a clinical statement receiving a median score greater than 7 on a 9-point scale from the panel was regarded as valid. After evaluation by the moderators, the amendment of the Japanese consensus guidelines for AIP has been proposed in 2020.

## Introduction

Since Yoshida et al. first proposed the concept of autoimmune pancreatitis (AIP) in 1995 [1], AIP has been accepted worldwide as a distinctive type of pancreatitis [1–5]. The Japan Pancreas Society (JPS) and the Research Committees for Intractable Pancreatic Disease supported by the Ministry of Health, Labour and Welfare of Japan (MHLWJ) proposed the Japanese consensus guidelines for the management of AIP in 2009 [6–8] and updated them in 2013 [9–11]. As the number of publications on AIP extracted from the PubMed and Cochrane Library databases increased from 1,843 in 2013 to 5,218 in 2019, the Japanese consensus guidelines need to be revised. Most of the evidence levels of the specific clinical statements were still lower than grade III proposed by the Agency for Health Care Policy and Research in 1993. Therefore, we have developed the revised version of the consensus guidelines according to the modified Delphi approach [6–12]. During the first phase, 20 specialists (17 pancreatologists, one radiologist, one respiratory system expert, and one pathologist) in the members of the Research Committees for IgG4-related disease supported by MHLWJ revised the 39 clinical questions (CQs) and statements for (i) concept and diagnosis (14CQS), (ii) extra-pancreatic lesions (9 CQs), (iii) differential diagnosis (5 CQs), and (iv) treatment (11 CQs) based on the selected papers as described above.

In the second phase, the expert ten panelists individually rated these clinical statements on a 9-point scale for appropriateness, and discussed areas of disagreement and uncertainty [6–12]. A clinical statement receiving a median score greater than 7 from the panel was regarded as valid. During the third phase, the revised clinical statements were rated again. Based on the two-round modified Delphi approach, guideline statements for diagnosis and management of AIP were developed. Finally, the evaluation committee comprised three moderators and 17 special evaluating members selected from JPS evaluated all the clinical questions, statements, and descriptions. In the revised consensus-based guidelines, the statements for clinical practice were evaluated as “strongly recommendable” (level A) or “strongly unrecommendable (level D)” for receiving score of 9, and “ordinarily recommendable” (level B), or “unrecommendable” (level C) for that less than 9 according to the grading proposed by U.S. Preventive Services Task Force[13].

The Japanese language full versions of the present guidelines have been published in the official journal of the Japan Pancreas Society, “Suizo” in 2020[14]. The English digestive version is scheduled to be published in the Journal of Gastroenterology with approval from both of Professor Sata, the Editor-in-Chief of “Suizo”, and Professor Seno, the Editor-in-Chief of the Journal of Gastroenterology.

## Clinical questions and statements

### I. Concept and diagnosis

#### CQ-I-1) What is “autoimmune pancreatitis (AIP)”?

- Autoimmune pancreatitis (AIP) is a distinct form of pancreatitis characterized clinically by frequent presentation with obstructive jaundice, with or without a pancreatic mass; histologically by a lymphoplasmacytic infiltrate and fibrosis; and therapeutically by a dramatic response to glucocorticoids.
- AIP is classified as two subtypes, type 1 and type 2 AIP. As most cases of AIP in Japan are type 1, the simple term of “AIP” usually means type 1 AIP in Japan.
- Type 1 AIP shows lymphoplasmacytic sclerosing pancreatitis (LPSP) characterized by massive infiltration of lymphocytes and plasmacytes, especially IgG4-positive plasmacytes, storiform fibrosis, and obliterative phlebitis. It is a pancreatic manifestation of a systemic disorder, IgG4-related disease (IgG4-RD).
- Type 2 AIP, idiopathic duct-centric pancreatitis (IDCP) or AIP with granulocyte epithelial lesions (GEL), commonly observed in Europe and the United States, shows neutrophilic lesions and therefore is a different condition than type 1 AIP.

 < Description > 

Patients with type 1 AIP occasionally have extra-pancreatic lesions such as sclerosing cholangitis, dacryoadenitis, sialadenitis, retroperitoneal fibrosis, or nephritis, all of which show similar pathological findings [1–5].

#### CQ-I-2) Are there characteristic clinical symptoms in AIP?

- No specific symptoms are presented by patients with type 1 AIP. In fact, these patients show no specific symptoms, but may have minor abdominal pain, obstructive jaundice, and symptoms of diabetes mellitus and/or accompanying extra-pancreatic lesions.

 < Description > 

The majority of the symptoms in type 1 AIP are associated with sclerosing cholangitis, diabetes mellitus, dacryosialoadenitis, retroperitoneal fibrosis, obstructive jaundice, polydipsia/polyuria, or general fatigue, and xerostomia/xerophthalmia or hydronephrosis, but rare fever [15]. Patients with type 2 AIP commonly develop abdominal pain similar to that in acute pancreatitis [16].

#### CQ-I-3) How is AIP found?

- The majority of AIP patients see doctors with complaints of minor abdominal pain, general malaise, jaundice, or dry mouth (Level of recommendation: B).
- AIP is often found in patients who have elevated levels of hepatobiliary enzymes, obstructive jaundice, or worsened diabetes mellitus during examinations for differential diagnosis from pancreatic and biliary cancers (Level of recommendation: B).
- Enlarged pancreas demonstrated by abdominal ultrasonography often leads to the detection of AIP (Level of recommendation: B).

 < Description > 

AIP is often found in patients who presented pancreatic enlargement on imaging modalities such as ultrasound, CT, or MRI during examinations for differential diagnosis from pancreatic and biliary cancers, or extra-pancreatic lesions and inflammatory bowel diseases[15–18].

#### CQ-I-4) What are the characteristics of blood-biochemical and immunological findings in AIP?

- Although there are no disease-specific serum biochemical findings associated with AIP, increased serum levels of pancreatic enzymes, hepatobiliary enzymes, and total bilirubin have been commonly recorded in AIP (Level of recommendation: A)
- Serum levels of IgG4 have the highest diagnostic value as a single serological diagnostic method among all available ones; however, this test is also not specific to the disease (Level of recommendation: A).

 < Description > 

Most of the cases show increased hepatobiliary enzymes (60–82%) and total bilirubin (39–62%) [15–19]. The occurrence rate of abnormal levels of serum pancreatic enzymes is lower in AIP (36%–64%) compared with that in acute pancreatitis or acute exacerbation of chronic pancreatitis [15–19]. The immunological abnormalities include increased peripheral eosinophils (38%), high incidences of hypergammaglobulinemia (43%), increased serum IgG (58.5–80%)/IgE levels (76%), rheumatoid factor (25%), and autoantibodies such as antinuclear (40–64%) [15–19], anti-carbonic anhydrase II (55%) [19], anti-lactoferrin (75%) [19], anti-annexin-A11(18%) [20], anti-laminin511 (51%) [21, 22], and anti-galectin-3 (28%) antibodies [23, 24], but anti-SSA/B or antimitochondrial antibodies are extremely rare [15–19]. The increased serum levels of IgG4 (68–92%) have the highest diagnostic value with the disease sensitivity of 80% and specificity of 98% when compared with those for pancreatic cancer, but is not a specific marker [15–19].

#### CQ-I-5) Are there pancreatic exocrine and endocrine dysfunctions?

- AIP is often associated with pancreatic exocrine and endocrine dysfunctions. Occurrence ratios are about 80% and 70% for exocrine and endocrine dysfunctions, respectively (Level of recommendation: A).

 < Description > 

Abnormal pancreatic exocrine function has been reported in 81–88% of the patients with AIP by the BT-PABA (PFD test), an examination of fecal elastase 1 [25], or secretion dysfunction, and diabetes mellitus in 42–78% [26–29]. Additionally, 49% of diabetes mellitus patients who developed AIP simultaneously were reported to need insulin therapy [30].

#### CQ-I-6) What are the characteristic findings of abdominal ultrasonography in AIP?

- Ultrasonic findings in patients with AIP are characterized by a diffusely enlarged pancreas with low echo; ‘‘sausage-like’’ pancreas (Level of recommendation: A).
- A focally enlarged pancreas must be distinguished from pancreatic cancer through a differential diagnosis (Level of recommendation: A).

 < Description > 

No dilatation of the main pancreatic duct (MPD) is seen in most cases of diffusely enlarged pancreas with ‘‘sausage-like’’ appearance. The enlarged area shows a low-echo image, in some cases with scattered high-echo spots [31, 32]. A focally enlarged pancreas must be distinguished from pancreatic cancer. Duct penetration may be a useful sign to rule out pancreatic cancer [33]. In some patients with AIP, ultrasonography shows a wall thickening of the bile duct and/or the gallbladder. A thickened bile duct wall is characterized by layered or parenchymal low-echo wall thickening.

#### CQ-I-7) What are the characteristic findings of abdominal computed tomography (CT) in AIP?

- Abdominal CT images of patients with AIP show a diffusely or locally enlarged pancreas (Level of recommendation: A).
- If a capsule-like rim or a distinctive delayed enhancement pattern on the dynamic CT is observed, AIP is highly suspected (Level of recommendation: A).

 < Description > 

AIP is hypovascular on the pancreatic phase and homogeneously enhanced on the delayed phase of the dynamic CT [31, 32, 34]. Delayed enhancement is one of the specific findings of AIP and is useful for differentiation between AIP and pancreatic cancer, but not seen in AIP when the amount of fibrosis is low. A capsule-like rim is seen as a band-like hypodense/hypointense area and is gradually enhanced on the dynamic CT [31, 32, 34]. The capsule-like rim was reported to reflect dense fibrosis around the lesion, although its frequency varied among different reports [31, 32, 34]. The finding is never seen in diseases other than AIP and is one of the specific findings that can differentiate AIP from pancreatic cancer.

#### CQ-I-8) What are the characteristic magnetic resonance (MR) findings? Can magnetic resonance cholangiopancreatography (MRCP) evaluate narrowing of the main pancreatic duct (MPD) in AIP?

- MR findings characteristic of AIP are a diffuse enlarged pancreas, shown as a low signal intensity on fat-suppressed T1-weighted imaging (FS-T1WI); speckled/dotted enhancement; a capsule-like rim on the pancreatic phase of dynamic contrast-enhanced MRI (DCE-MRI); and delayed enhancement on the delayed phase of DCE-MRI (Level of recommendation: A).
- At this moment, MRCP is not recommended for the accurate evaluation of the narrowing of the MPD (Level of recommendation: B).

 < Description > 

**Fig. 1:**
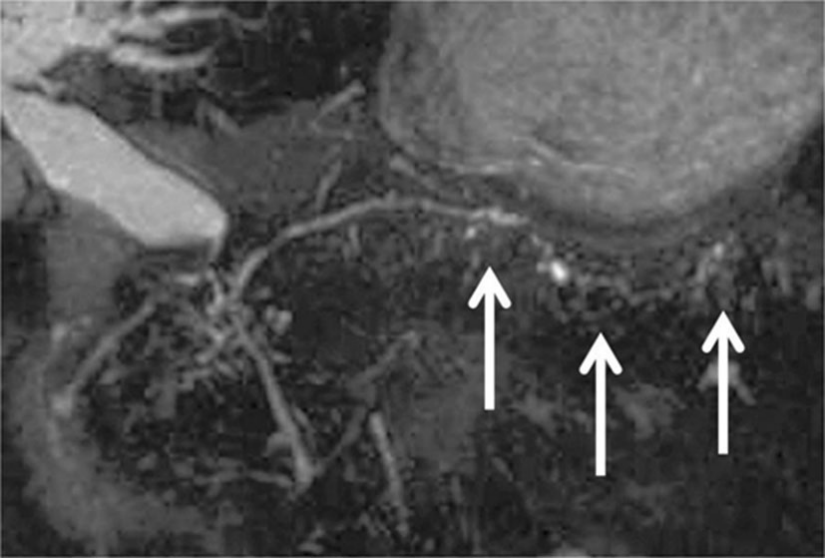
3D MRCP in AIP. 3D MRCP shows narrowing of the main pancreatic duct in the pancreatic body and tail (arrows)

A signal decrease on FS-T1WI and a signal increase on T2-weighted imaging are not a specific finding of AIP [31, 34]. Useful CT and MR findings to differentiate AIP from pancreatic cancer are also described in CQ-III-3. Although image resolution of MRCP is lower than that of endoscopic retrograde pancreatography (ERP), MRCP was adopted as a modality that could evaluate the pancreatic duct in the Japanese Clinical Diagnostic Criteria for Autoimmune Pancreatitis 2018 [35], because image quality of MRCP has been improved by the advancement of technology (Fig. 1) [34, 36–38].

#### CQ-I-9) What are the characteristic findings of fluorodeoxyglucose-positron emission tomography (FDG-PET) and gallium-scintigram in AIP?

- Patients with AIP display accumulation of gallium citrate (Ga-67) and FDG in pancreatic and extra-pancreatic lesions, which disappear shortly after glucocorticoid therapy. The characteristic accumulation patterns and kinetics of Ga-67 and FDG following glucocorticoids can be used for AIP diagnosis (Level of recommendation: B).

 < Description > 

Ga-67 accumulation is found in approximately 70% of pancreatic lesions [39], and its distribution and kinetics after steroid treatment can be used for the diagnosis of AIP. High accumulation of FDG is also observed in pancreatic and extra-pancreatic lesions [40–43]. The distribution pattern and kinetics of FDG after glucocorticoids are useful in distinguishing AIP from pancreatic cancer [44]. A steroid trial should be carefully performed at a specialized hospital after a negative work-up of malignancy. FDG-PET for AIP is not supported by Japanese medical insurance.

#### CQ-I-10) What are the characteristic findings of endoscopic retrograde cholangiopancreatography (ERCP) in AIP?

- ERCP shows narrowing of the MPD, which is characteristic of AIP (Level of recommendation A).
- AIP may be associated with stenosis of the bile duct (Level of recommendation A).

 < Description > 

The narrowing of the MPD is “unlike the obstruction or stenosis, as the narrowing extends to certain degree and the duct diameter is smaller (narrower) than normal, with some irregularities” [45, 46]. The typical case exhibits narrowing over one-third of the entire pancreatic duct. In most cases, no significant dilatation is observed above the narrowed area upstream of the MPD. If the narrowing is localized, it is necessary to consider differentiating the disease from pancreatic cancer. Typical pancreatic duct features of AIP, such as a side branch arising from the narrowed portion or multiple stenosis (“skip”) of the MPD, are useful for differential diagnosis from pancreatic cancer [4, 47–52]. Some AIP cases show multiple stenotic (“skip”) lesions. About 80% of patients with AIP show stenosis of the bile duct, most commonly in the distal region [53–55].

#### CQ-I-11) How is the pathology specimen collected?

- For cases in which a diagnosis cannot be made without pathology specimen collection, or cases in which malignancy is suspected or cannot be ruled out, specimen collection by endoscopic ultrasound-fine-needle aspiration (EUS-FNA) or endoscopic ultrasound-fine-needle biopsy (EUS-FNB) should be considered (Recommendation level A).
- Biopsy of the papilla of Vater may be added if ERCP is performed in the diagnostic process (Recommendation level B).

 < Description > 

EUS-FNA for AIP may be useful for distinguishing from malignancies, classification of AIP subtypes, and diagnosis in combination with other clinical findings. Two prospective studies of EUS-FNA for AIP reported that the diagnosis rate of ICDC level 1 was 0% and 43.4%, and that of level 2 was 68% and 15.1% [56, 57]. Recently developed core needles enable the collection of a large amount of tissue samples. In two prospective studies of EUS-FNB using the Franseen needle for AIP, ICDC histological findings of lymphoplasmacytic infiltration were observed in 84% and 100%, obliterative phlebitis in 24% and 43.6%, storiform fibrosis in 56% and 72.7%, and abundant IgG4-positive cells in 76% and 65.5% of the cases [55, 59]. Furthermore, the diagnosis rate of ICDC level 2 or higher was 78% and 92.7% [60].

#### CQ-I-12) What are the characteristic histopathological findings in AIP?

- AIP can be diagnosed when findings such as marked lymphoplasmacytic infiltration, numerous IgG4-positive plasma cells, storiform fibrosis, obliterative phlebitis, and inflammatory cell infiltration around the duct epithelium are present histologically (Level of recommendation: A)

 < Description > 

The presence of numerous IgG4-positive plasma cells is characteristic but not specific. Storiform fibrosis is a swirling pattern of inflammation containing lymphoplasmacytic infiltration, spindle-shaped cells, and variable degrees of fibrosis. Obliterative phlebitis is a venous stenosis or occlusion with AIP lesion involvement. Some recent studies indicate that an AIP diagnosis can be rendered by endoscopic ultrasound-guided fine-needle biopsy with a 22-gauge needle [56, 57, 60–62].

#### CQ-I-13) How to diagnose AIP?

- A comprehensive diagnosis must be performed based on pancreatic image findings, serological findings, histopathological findings, other organ involvements, and steroid effects (Level of recommendation: A)
- The ICDC for AIP can diagnose both type 1 and type 2 AIP (Level of recommendation: A)
- In Japan, most cases of AIP can be diagnosed by the Japanese Clinical Diagnostic Criteria 2018 (JPS-2018) for type 1 AIP (Level of recommendation: A)

 < Description > 

Based on the Japanese conditions, the JPS and the RCIPD-MLHWJ revised them into the clinical diagnostic criteria for AIP in 2018 [35] (Table 1).

**Table 1:**
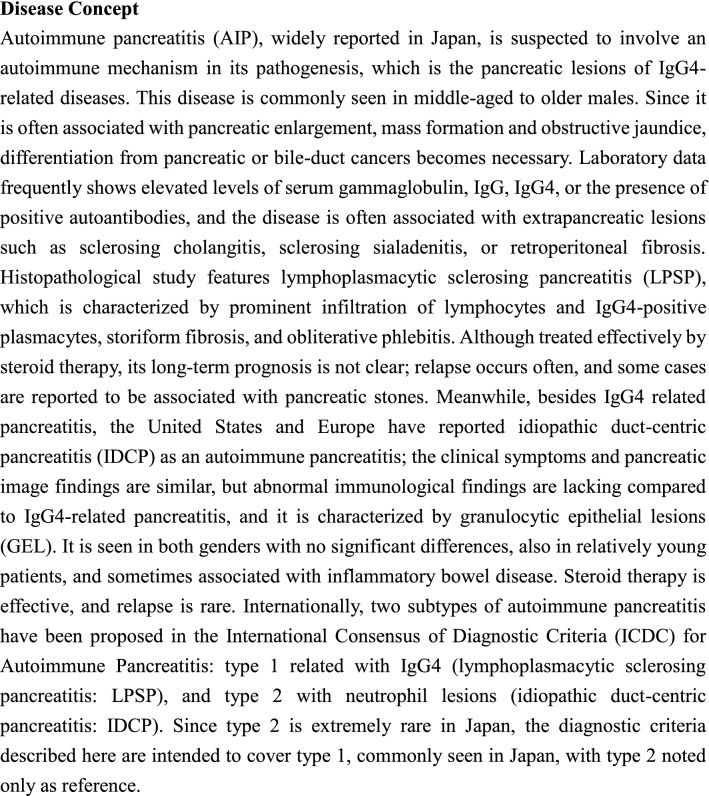

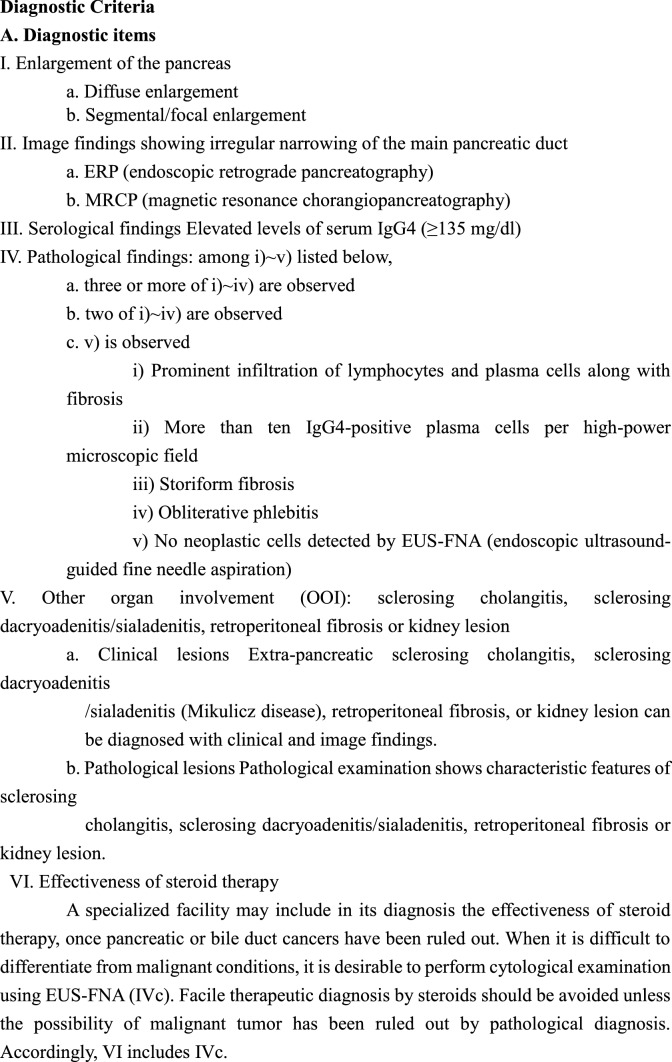

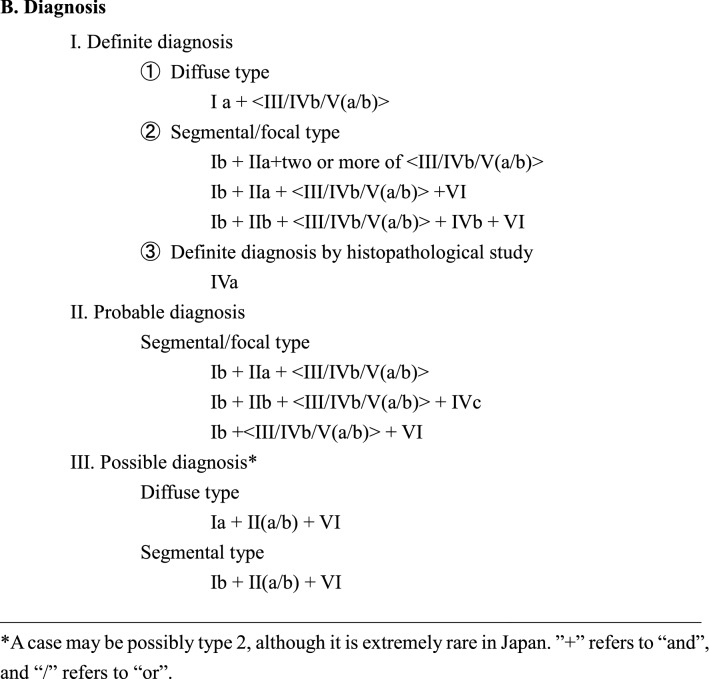
Japanese Clinical Diagnostic Criteria for Autoimmune Pancreatitis, 2018

#### CQ-I-14) Can AIP be diagnosed based on response to glucocorticoids?

- Response to glucocorticoids indicates possible AIP. However, response to glucocorticoids does not exclude the possibility of pancreatic cancer (Level of recommendation: B).
- In the cases of segmental/focal swelling or tumor-forming pancreas, glucocorticoid therapy should be performed only after a negative work-up for malignancy using ERCP or EUS-FNA cytology (Level of recommendation: B).

 < Description > 

Different from the previous Japanese diagnostic criteria for AIP 2002 [3] and 2006 [4], those for AIP 2018 adopted a response to glucocorticoids as a diagnostic criterion [35].

### II. Extra-pancreatic lesions

#### CQ-II-1) What types of extra-pancreatic lesions are associated with AIP?

- A variety of extra-pancreatic lesions are reportedly associated with AIP. Among those cited, closely associated lesions include lacrimal and salivary gland lesions, ophthalmic disease, respiratory lesions, bile duct lesions, kidney disease, periaortitis, and retroperitoneal fibrosis (Level of recommendation: B).

 < Description > 

Extra-pancreatic lesions related to AIP are prevalent in systemic organs [62] and share the same pathological conditions with favorable response to glucocorticoids, indicating a common pathophysiological background with IgG4-RD [64]. Extra-pancreatic lesions sometimes mimic primary lesions in the corresponding organ; however, recognition of these should aid in the accurate diagnosis of AIP [65].

#### CQ-II-2) Based on what findings are extra-pancreatic lesions of AIP diagnosed?

- The diagnosis of extra-pancreatic lesions associated with AIP is indicated when the lesions exhibit the characteristic clinical findings of IgG4-RD. The diagnosis of extra-pancreatic lesions can be made when the lesions fulfill the comprehensive diagnostic criteria for IgG4-RD or the corresponding organ-specific diagnostic criteria, although special attention should be paid for distinct differentiation from similar lesions due to other causes in the corresponding organ (Level of recommendation: B)

 < Description > 

Since extra-pancreatic lesions associated with AIP are part the characteristics of IgG4-RD, a diagnosis can be made when the lesions fulfill the comprehensive diagnostic criteria for IgG4-RD [66].

#### CQ-II-3) What are the differences between lacrimal and salivary gland lesions associated with AIP and those associated with Sjo¨gren’s syndrome?

- AIP-associated lacrimal and salivary gland lesions usually persist for over 3 months and show symmetrical distribution without pain, whereas the salivary gland lesions in Sjo¨gren’s syndrome sometimes display repetitive and unilateral distribution and pain and frequently subside spontaneously (Level of recommendation: B)
- Compared with those of Sjo¨gren’s syndrome, AIP-associated lacrimal and salivary gland lesions exhibit normal or slightly impaired exocrine function, presenting as a slight or negligible dryness in the eyes and mouth (Level of recommendation: B)
- Compared with those of Sjo¨gren’s syndrome, AIP-associated lacrimal and salivary gland lesions show negative results in tests for SS-A/Ro and SS-B/La autoantibodies (Level of recommendation: A)
- Unlike those of Sjo¨gren’s syndrome, AIP-associated lacrimal and salivary gland lesions respond favorably to glucocorticoids (Level of recommendation: A)

 < Description > 

AIP-associated lacrimal and salivary gland lesions are now recognized as IgG4-RD, IgG4-related dacryoadenitis, and sialadenitis [58]. Useful clinical findings have been reported for the distinction between AIP-associated lacrimal and salivary gland lesions and those associated with Sjo¨gren’s syndrome [67–69]. The former represents a highly active state, with raised serum IgG4 concentrations and more severe pancreatic swelling [70, 71].

#### CQ-II-4) What kind of respiratory lesions are associated with AIP?

- Respiratory lesions associated with AIP include bronchial asthma (or asthmatic symptoms), interstitial lung diseases, inflammatory pseudotumor of the lung, thickening of the tracheal or bronchial wall and bronchial vascular bundle, pleural lesions, and hilar or mediastinal lymphadenopathy. The lesions must be differentiated from other interstitial lung diseases and tumors. The pathology of these lesions includes numerous IgG4-bearing plasma cell infiltrations and a favorable response to glucocorticoids (Level of recommendation: B)

 < Description > 

Respiratory involvement is seen in 13–54% of AIP patients [63, 72–74]. Some patients have asthmatic symptoms; however, 43–50% of the patients have few respiratory symptoms [72, 73]. Pathological examination shows lymphoplasmacytic infiltration with fibrosis in and around the lymphatic routes, with distribution well correlated with the radiological manifestations [75]. If glucocorticoid therapy is ineffective, other diseases such as progressive fibrosing interstitial lung diseases should be considered. Furthermore, surgical resection should be considered if lung cancer cannot be ruled out as the cause of the lung lesions [75].

#### CQ-II-5) How is AIP-associated sclerosing cholangitis (AIP-SC) differentiated from primary sclerosing cholangitis (PSC) or biliary malignancies?

- The differentiation between AIP-SC and PSC or biliary malignancies should be ascertained carefully based on a combination of clinical features, pathological findings, and imaging tests such as cholangiography, ultrasonography, endoscopic ultrasonography (EUS), intraductal ultrasonography (IDUS), CT, and MRI (Level of recommendation: A)
- Clinical findings of extra-pancreatic lesions specific for IgG4-RD support the diagnosis of IgG4-SC (Level of recommendation: A)

 < Description > 

AIP-SC, also known as IgG4-SC, characteristically displays lower (intrapancreatic) bile duct stenosis, although it may also exhibit restricted stenosis from the hilar to the extrahepatic bile ducts or multiple stenotic regions in the intrahepatic bile ducts [76]. The lower bile duct lesions must be distinguished from pancreatic cancer or common bile duct cancer; intrahepatic and hilar bile duct lesions should be differentiated from PSC and cholangiocarcinoma, respectively. There are several key differences between IgG4-SC and PSC [77, 78]. IgG4-SC sometimes shows slight or no pancreatic lesions, which may lead to a misdiagnosis of PSC [79, 80]. IgG4-SC with localized bile duct stenosis must be clearly differentiated from bile duct cancer. Since it can be difficult for cholangiography alone to distinguish between these conditions, careful examination with other tests is necessary [81]. When diagnosing IgG4-SC, it is recommended to refer to the clinical diagnostic criteria for IgG4-SC [82] and the clinical practice guidelines for IgG4-SC [83].

#### CQ-II-6) What IDUS findings are characteristics of IgG4-related SC?

- IDUS shows symmetrical thickening of the entire circumference of affected stenotic bile duct walls, with a smooth inner surface and outer margin and homogeneous inner zone (Level of recommendation: B).
- IDUS discloses widespread wall thickening of the bile duct, whereas cholangiography shows no stricture (Level of recommendation: B).

 < Description > 

**Fig. 2:**
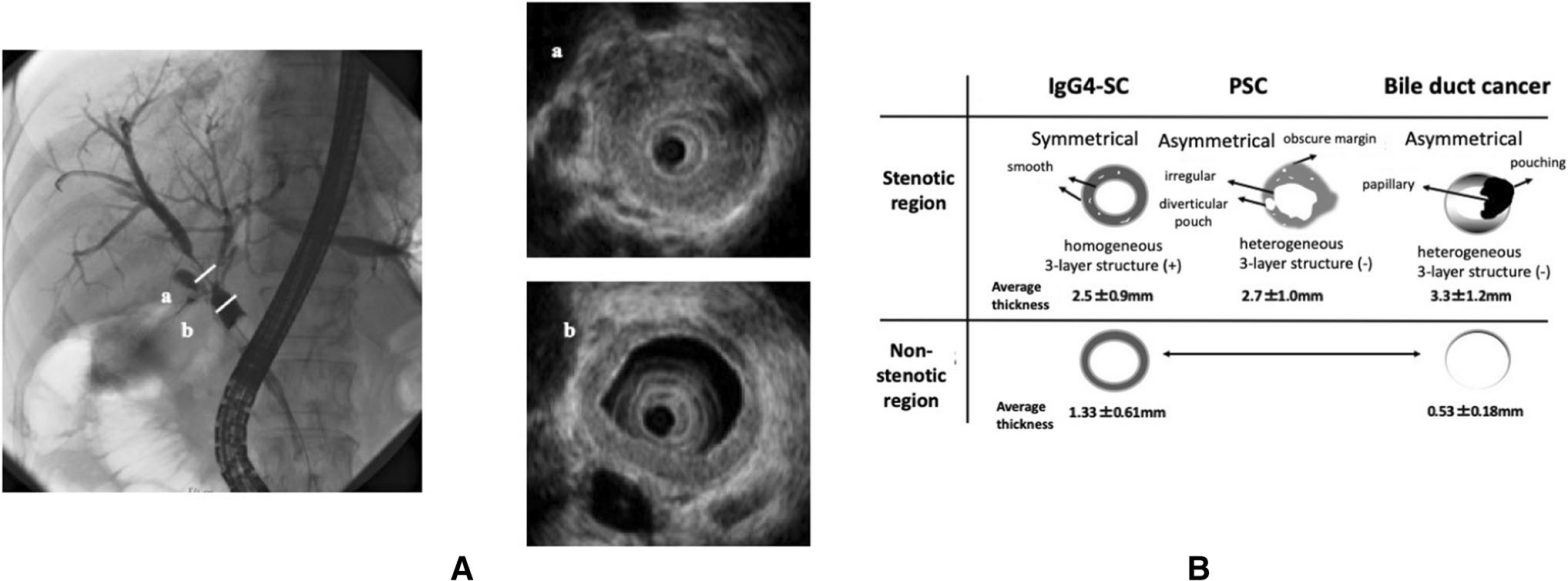
Cholangiography and IDUS findings of IgG4-related sclerosing cholangitis. **A** Cholangiography reveals hilar stenosis. **a**. IDUS findings at a stenotic region showing entire circumferential and symmetrical wall thickening and homogeneous inner zone. **b** IDUS findings at a non-stenotic region showing similar wall thickening and homogeneous inner zone, with the smooth inner surface and outer margin observed in the stenotic region. **B** Differentiation by IDUS findings. In IgG4-SC, IDUS shows preservation of the three-layer structure, symmetrical thickening of the entire circumference of the wall, and homogeneous inner zone at affected stenotic regions. Even at non-stenotic regions, similar wall thickening and homogeneous inner zone can be seen. In PSC, IDUS displays asymmetrical wall thickening with irregular inner surface and interrupted outer margins, heterogeneous inner zone, destruction of the three-layer structure, and a diverticular-like pouch. In bile duct cancer, IDUS shows asymmetrical wall thickening with irregular inner surface and interrupted outer margin according to cancer invasion as well as heterogeneous inner zone. Unlike IgG4-SC, there is no wall thickening at non-stenotic regions

Different from IDUS findings at affected stenotic regions on cholangiography in IgG4-SC, that of bile duct cancer exhibits asymmetrical thickening of the entire circumference of bile duct walls, with an interrupted outer margin and irregular inner surface and heterogeneous inner zone (Fig. 2A) [84, 85]. In AIP, lower bile duct stenosis is supposedly caused by two mechanisms, extrinsic compression by the swollen pancreas head and thickening of the bile duct wall found in IgG4-SC, while only extrinsic compression is present in pancreatic cancer [86, 87]. PSC is another mimic of IgG4-SC, presenting the IDUS findings of asymmetrical wall thickening, irregular inner surface, interrupted outer margin, heterogeneous inner zone, and the destruction of the three-layer structure, all of which set it apart from IgG4-SC (Fig. 2B) [88, 89].

#### CQ-II-7) How can retroperitoneal lesions associated with AIP be diagnosed as AIP-associated retroperitoneal fibrosis (AIP-RF)?

- CT and MRI are commonly used to detect the morphological findings characteristic of RF associated with AIP. These findings include soft-tissue densities that represent masses around the ureter and aorta, near the vertebrae, or in the pelvic cavity (Level of recommendation: B).

 < Description > 

**Fig. 3:**
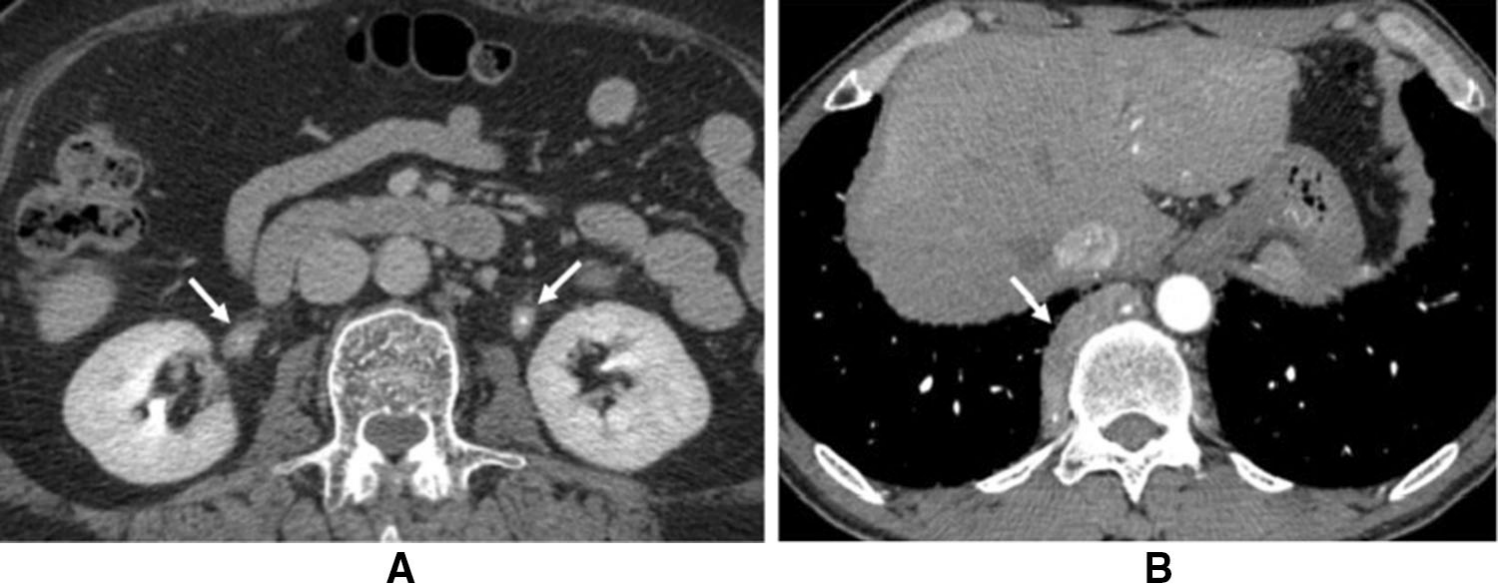
Enhanced CT images of AIP-related retroperitoneal fibrosis. **A** Enhanced CT (late phase) shows soft-tissue masses around both ureters (arrows). **B** Enhanced CT (arterial phase) reveals a soft-tissue mass anterior to the vertebra (arrow)

AIP-RF is characterized by morphological findings detected by CT and MRI analyses (Fig. 3A,B) [64, 90]. In addition, intense FDG uptake is typically observed in corresponding lesions on FDG-PET [91]. Histological studies of biopsy specimens revealed numerous IgG4-bearing plasma cell infiltrations and obstructive phlebitis [64, 90]. Soft-tissue masses around the ureter sometimes induce ureteral strictures, which may result in hydronephrosis and irreversible renal failure [92]. These lesions typically respond favorably to glucocorticoids [64].

#### CQ-II-8) How can renal lesions associated with AIP be diagnosed as AIP-associated kidney disease (AIP-KD)?

- AIP-KD is referred to as IgG4-KD, with most lesions displaying tubulointerstitial nephritis (Level of recommendation: B).
- Dynamic contrast-enhanced CT shows poorly enhanced multiple nodules, wedge-shaped lesions, and/or round lesions in the renal cortex, renal swelling or masses, and mass lesions in the renal pelvis (Level of recommendation: B).

 < Description > 

**Fig. 4:**
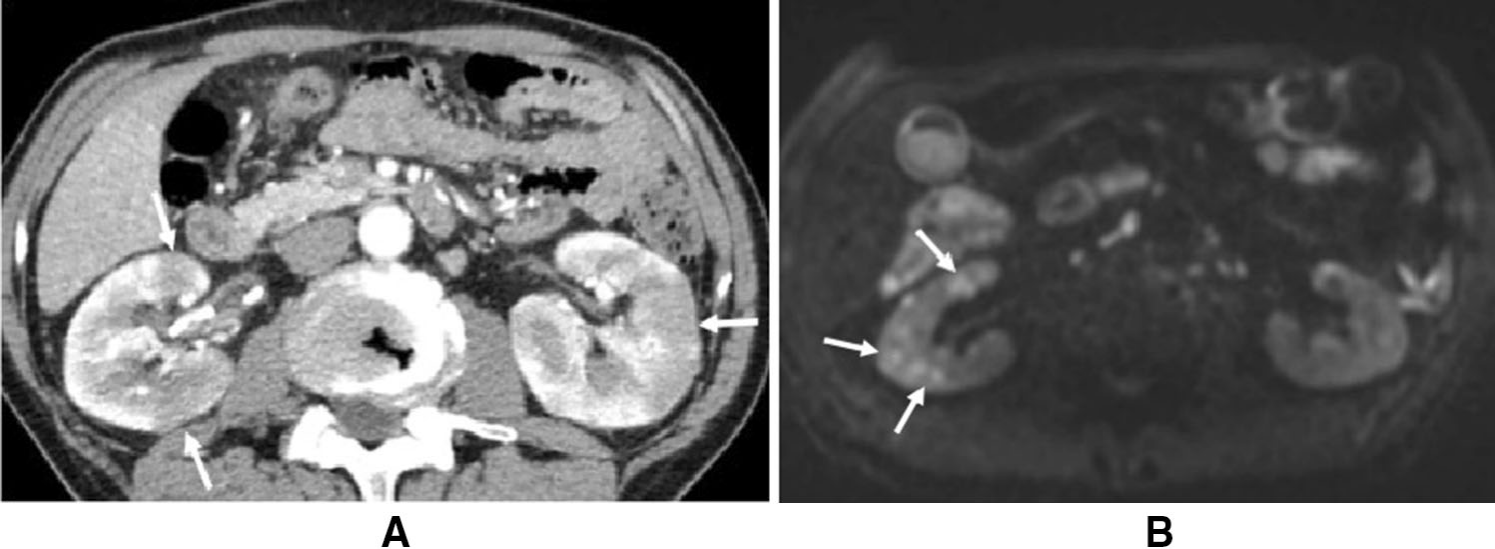
CT/MRI images of AIP-related kidney disease (AIP-KD). **A** Dynamic contrast-enhanced CT (arterial phase) shows multiple poorly enhanced nodules in both renal cortexes (arrows). **B** Diffusion-weighted MRI reveals multiple decreased diffusion areas in the right kidney (arrows)

AIP-KD is also known as IgG4-KD and diagnosed by the diagnostic criteria for IgG4-KD with several characteristic image findings (Fig. 4A,B [64, 93, 94]. As most lesions are considered tubulointerstitial nephritis [95, 96], proteinuria is usually negative or mild. Clinically, some patients show acute or progressive renal failure, while others are diagnosed through imaging abnormalities with normal or slightly decreased renal function. AIP-KD seldom shows glomerular lesions, while membranous nephropathy is the most common glomerular disease [97].

#### CQ-II-9) How can aortic lesions associated with AIP, IgG4-related periaortitis/periarteritis be diagnosed and managed?

- Characteristic image findings of AIP-associated aortitis are thickening of the aortic wall or soft-tissue mass around the aorta, sometimes as an aneurysm (Level of recommendation: B).
- Since some cases are complicated with aneurysm rupture during glucocorticoid therapy, surgery should be considered based on the morphology and chronological changes of aneurysmal conditions (Level of recommendation: B).

 < Description > 

**Fig. 5:**
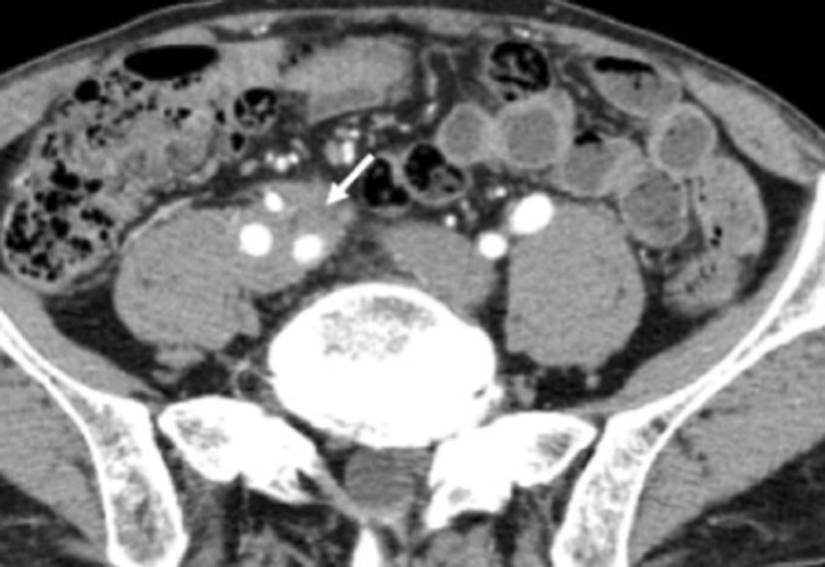
Contrast-enhanced CT images of IgG4-related periaortitis/periarteritis. Contrast-enhanced CT (aortic phase) shows soft-tissue mass (arrow) around the right iliac artery

AIP-associated aortitis shows systemic distribution and is now recognized as IgG4-related periaortitis/periarteritis [98–100], although most cases exhibit continuous distribution from the abdominal aorta to the iliac artery [101], whereby this lesion had been included in retroperitoneal fibrosis. Symptoms such as fever and pain may be observed, but most of the cases are asymptomatic. Image findings show soft-tissue mass around the aorta or arteries (Fig. 5), and characteristic pathological findings are found at the adventitia and surrounding adipose tissue [102]. These lesions respond well to glucocorticoids, and are differentiated from infectious aortic aneurysm, aortitis syndrome, malignant tumors, and chronic periarteritis caused by drugs. As aneurysm rupture is possible, special attention to morphology and chronological changes in aneurysmal conditions during glucocorticoid therapy is needed [103, 104].

### III. Differential diagnosis

#### CQ-III-1) What clinical symptoms or findings are useful for differentiating between AIP and pancreatic cancer?

- Severe abdominal pain and obstructive jaundice unresponsive to glucocorticoids suggest the possibility of pancreatic cancer, whereas characteristic extra-pancreatic lesions associated with IgG4-RD indicate AIP (Level of recommendation: B).

 < Description > 

In AIP, abdominal pain is mild and the jaundice occasionally fluctuates, spontaneously subsides, and responds well to glucocorticoids. Various extra-pancreatic lesions associated with IgG4-RD can be found in AIP [1, 7, 10, 65].

#### CQ-III-2) Can pancreatic cancer be ruled out if serum IgG4 level is high?

- Serum IgG4 level is a useful serum marker for the diagnosis of Type I AIP. However, high IgG4 levels are also sometimes seen with pancreatic cancer, so pancreatic cancer cannot be ruled out with high serum IgG4 levels alone (Recommendation: B).

 < Description > 

Serum IgG4 level is useful for differentiating between Type I AIP and pancreatic cancer. The American College of Rheumatology (ACR)/European League Against Rheumatism (EULAR) Classification criteria for IgG4-RD established in 2019 stated that the possibility of type I AIP is extremely high if serum IgG4 levels are five or more times higher than the upper limit of normal [105].

#### CQ III-3) What are the useful CT, MR, and FDG-PET findings for differentiating AIP from pancreatic cancer?

- In AIP patients, a straightened margin of the pancreas and capsule-like rim are sometimes seen as a characteristic finding (Level of recommendation: A).
- Speckled/dotted enhancement on the pancreatic phase of dynamic contrast-enhanced CT/MRI (DCE-CT/MRI) and homogeneous delayed enhancement on the delayed phase of DCE-CT/MRI are characteristic features of AIP and are useful for differentiation from pancreatic cancer (Level of recommendation: A).
- In AIP patients, a duct-penetrating sign is occasionally seen on T2-weighted imaging or MRCP, which is almost never observed in pancreatic cancer (Level of recommendation: A).
- Although a locally enlarged AIP lesion is sometimes difficult to differentiate from pancreatic cancer, AIP enlargement is improved by glucocorticoid therapy (Level of recommendation: A).
- FDG uptake is frequently seen in AIP patients. Diffuse/multi-focal uptake in pancreatic lesions as well as uptake in extra-pancreatic lesions including lachrymal/salivary glands and hilar lymph nodes are useful for differentiation from pancreatic cancer (Level of recommendation: C).

 < Description > 

**Fig. 6:**
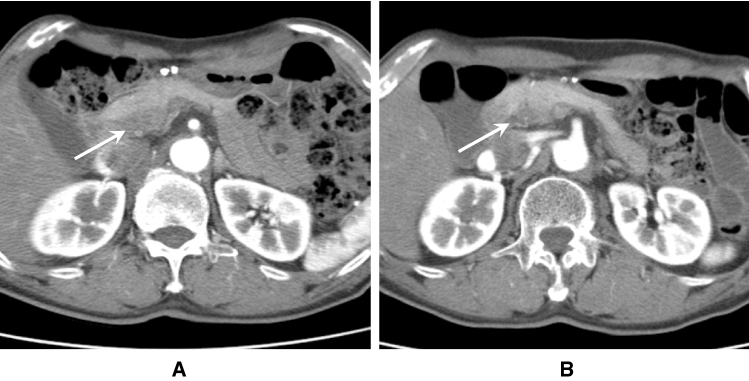
Contrast-enhanced CT images of focal AIP before and after corticosteroid therapy. **A** DCE-CT (pancreatic phase) shows a hypovascular lesion in the pancreatic head (arrow). **B** DCE-CT (pancreatic phase) after corticosteroid therapy reveals improvement of the pancreatic head lesion (arrow)

**Fig. 7:**
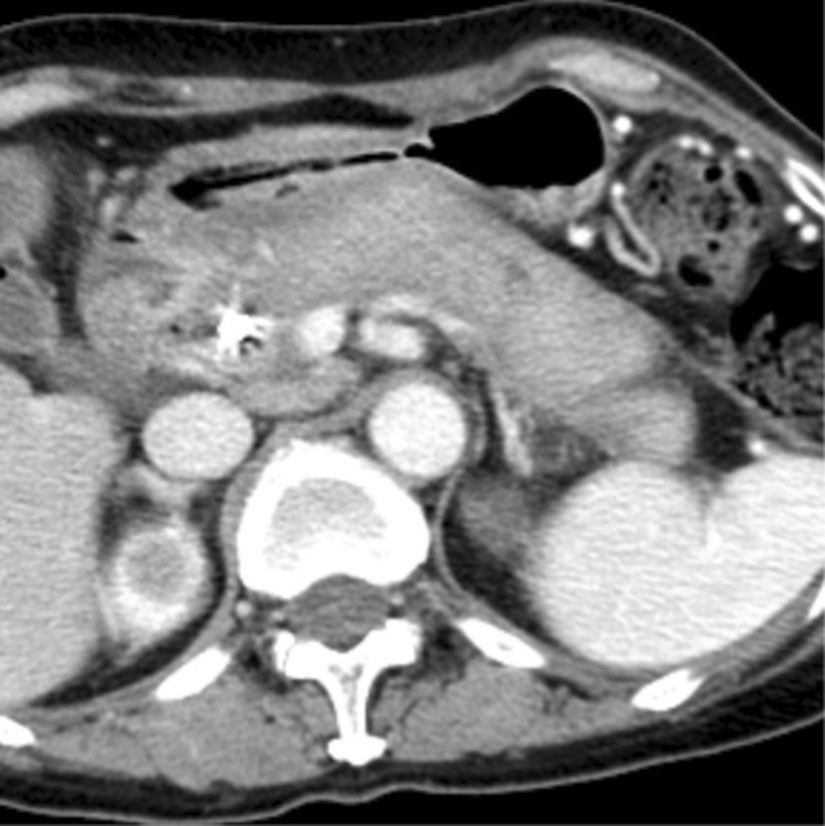
Contrast-enhanced CT images of AIP with diffuse pancreatic enlargement. DCE-CT (pancreatic phase) shows diffuse pancreatic enlargement, straightened pancreatic margin, and capsule-like rim

**Fig. 8:**
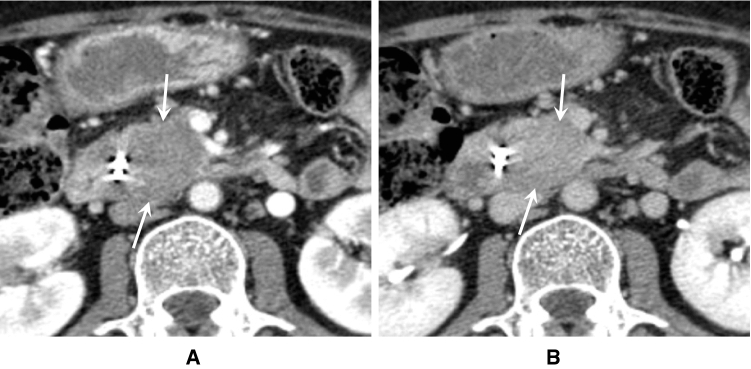
Contrast-enhanced CT images of AIP with focal pancreatic enlargement. **a** DCE-CT (pancreatic phase) shows a hypovascular lesion in the pancreatic head (arrows). **b** DCE-CT (delayed phase) depicts a pancreatic head lesion with homogeneous delayed enhancement (arrows)

**Fig. 9:**
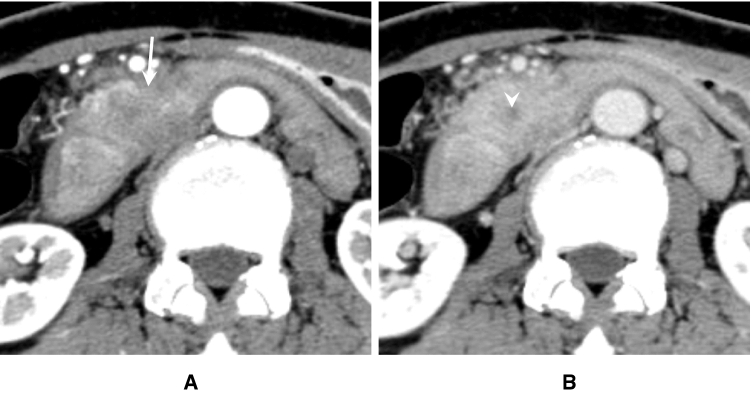
Contrast-enhanced CT images of pancreatic head cancer. **a** DCE-CT (pancreatic phase) shows a hypovascular lesion in the pancreatic head (arrow). **b** DCE-CT (delayed phase) reveals a pancreatic head lesion with inhomogeneous delayed enhancement (poor enhancement in the central area; arrowhead)

Although AIP sometimes displays hypovascular focal pancreatic swelling similar to that in pancreatic cancer, AIP lesions are improved with glucocorticoid therapy (Fig. 6 A,B). The margin of the pancreas is lobulated and the inside structure has a cobblestone appearance in elderly individuals. In contrast, the margin of the pancreas in AIP patients is straightened, with a “sausage-like” appearance (Fig. 7). For an accurate diagnosis, DCE-CT/MRI should be performed whenever possible. Useful findings for differentiation of AIP from pancreatic cancer include a duct-penetrating sign [106], speckled/dotted enhancement [107], capsule-like rim [31], and delayed homogeneous enhancement (Figs. 8, 9.) [46], since the specificity of those findings is high [34]. On MRCP, skipped narrowing and absent main pancreatic duct (MPD) dilatation have been also reported as characteristic AIP findings [34, 106]. Regarding diffusion-weighted imaging, the apparent diffusion coefficient (ADC) value is less useful for differentiation as different MR devices and scanning parameters have produced various cut-off values (Table 2) [34, 108–110]. Lastly, FDG-PET is of value for detecting AIP and extra-pancreatic lesions [40], with FDG uptake being decreased after corticosteroid therapy [43].

**Table 2:** Characteristic findings of focal AIP and pancreatic cancer

	Focal AIP	Pancreatic cancer
DCE-CT/MRI		
Pancreatic phase	Hypovascular (with speckled/dotted enhancement), capsule-like rim (hypovascular)	Hypovascular (non-specific)
Delayed phase	Homogeneous delayed enhancement	Inhomogeneous delayed enhancement (target pattern)
Fat-suppressed 1-weighted imaging	Hypointensity (with speckled/dotted hyperintensity)	Hypointensity (non-specific)
T2-weighted imaging	Homogeneous hyperintensity, duct-penetrating sign, capsule-like rim (hypointensity)	Inhomogeneous hyperintensity (target pattern)
MRCP	Skipped narrowing, duct-penetrating sign, no MPD dilatation	Marked MPD dilatation
Diffusion-weighted imaging	Hyperintensity (non-specific), low ADC value compared with that of pancreatic cancer (cut-off values are inconsistent, with some overlap)	Hyperintensity (non-specific)
FDG-PET	Diffuse or multi-focal uptake in the lesion, uptake in extra-pancreatic lesions (FDG-PET for AIP is not covered by national medical insurance in Japan)	Nodular uptake in the lesion (non-specific)

*ADC* apparent diffusion coefficient, *AIP* autoimmune pancreatitis, *DCE* dynamic contrast-enhanced, *MPD* main pancreatic duct, *FDG-PET* fluorodeoxyglucose-positron emission tomography, *MRCP* magnetic resonance choangiopancreatography

#### CQ-III-4) What EUS findings are useful for differentiating between AIP and pancreatic cancer or ordinary chronic pancreatitis?

- In AIP, typical EUS findings show a relatively homogeneously hypoechoic pancreas with a diffuse pattern and linear or reticular (tortoiseshell pattern) hyperechoic inclusions in the parenchyma along with a hypoechoic band in the periphery (Level of recommendation: B).
- Compared with chronic pancreatitis, EUS in AIP typically reveals a homogeneous hypoechoic pattern in the pancreatic parenchyma, rarely showing the characteristics of chronic pancreatitis (e.g., heterogeneous texture, lobule-shaped margin, and hyperechoic ductal margin) (Level of recommendation: B).
- EUS of localized masses in AIP also display hypoechoic patterns that mimic those of pancreatic cancer. Linear or reticular (tortoiseshell pattern) hyperechoic inclusions and duct penetration are useful signs for distinguishing AIP from pancreatic cancer (Level of recommendation: B).
- Contrast-enhanced EUS sometimes provides useful findings for the differentiation of AIP from pancreatic cancer (Level of recommendation: B).
- EUS with fine-needle aspiration (EUS-FNA) has diagnostic utility not only for ruling out pancreatic cancer, but also for diagnosing AIP (Level of recommendation: B).

 < Description > 

A localized mass with a hypoechoic pattern has been described in EUS studies of both AIP and pancreatic cancer, which requires further attention [111] using specific methods such as EUS-FNA [112]. EUS-FNA also provides useful information for the histological diagnosis of AIP [60, 113].

#### CQ-III-5) Is it possible to distinguish changes associated with pancreatic cancer from AIP histologically?

- When neutrophilic infiltrates, inflammatory infiltrates with edema in the lobules, proliferation of plump fibroblasts, and lymphocyte-predominant infiltrates are present in a biopsy specimen, pancreatic cancer cannot be excluded (Level of recommendation: C).
- A finding of numerous IgG4-positive plasma cells should not be used to distinguish between pancreatic cancer and AIP (Level of recommendation: B).

 < Description > 

To avoid a false-negative diagnosis of pancreatic cancer, caution must be paid notably in cases with neutrophilic infiltrates, inflammatory infiltrates with edema in the lobules, proliferation of plump fibroblasts, and lymphocyte-predominant infiltrates with scarce plasma cells, which are more common in pancreatic cancer than in AIP [10]. Numerous IgG4-positive plasma cells can be observed even in cases not otherwise typical of AIP [114, 115], although rare cases show concomitant pancreatic cancer and AIP [116, 117].

### IV. Treatment and prognosis

#### CQ-IV-1) Do AIP patients improve spontaneously?

- Some AIP patients improve spontaneously (Recommendation: None).

 < Description > 

Most AIP patients who improved spontaneously did not have bile duct stenosis and had less frequent elevation of serum IgG4 levels [118, 119]. Spontaneous improvement has been reported in 10% of non-jaundiced AIP patients [118] and in 55.7[17]  – 65%[120] of AIP patients without glucocorticoid therapy. Furthermore, in the 97 patients, female gender and stent placement for jaundice were identified as predictors of transient remission, and new-onset diabetes mellitus and the presence of extensive multi-organ involvement were identified as risks of relapse [17].

#### CQ-IV-2) What are the indications for glucocorticoid therapy in AIP patients?

- Indications for glucocorticoid therapy in AIP patients are symptoms such as obstructive jaundice, abdominal pain, back pain, and the presence of symptomatic extra-pancreatic lesions (Level of recommendation: A).

 < Description > 

According to a Japanese multicenter study [121], the remission rate of AIP (98%) in steroid-treated AIP was significantly higher than that (74%) without steroid therapy. The most common indication for steroid therapy was obstructive jaundice in the Japanese study [121] and an international survey [122], (60% and 63%, respectively). Persistent abdominal pain or back pain and associated symptomatic extra-pancreatic lesions such as retroperitoneal fibrosis, interstitial pneumonia, tubulointerstitial nephritis, hepatic or pulmonary pseudotumor, pachymeningitis, and pericarditis are indications for glucocorticoid therapy [121–123]. A facile steroid trial to differentiate AIP from pancreatic cancer should be avoided [35].

#### CQ-IV-3) How should initial glucocorticoid therapy be performed?

- Before glucocorticoid therapy, jaundice should be managed by biliary drainage in patients with obstructive jaundice, and blood glucose levels should be controlled in patients with diabetes mellitus. The recommended initial oral prednisolone dose for induction of remission is 0.6 mg/kg/day, which is administered for 2–4 weeks and then gradually tapered (Level of recommendation: B)

 < Description > 

Prior to initiating glucocorticoid therapy, it is important to distinguish AIP from pancreatic or biliary cancer with imaging studies and a pathological approach via endoscopy [121]. A Japanese nationwide survey (type 1 AIP; n = 563) revealed that glucocorticoid therapy achieved a high remission rate of 98% after 6.8 months and demonstrated the validity of the initial dose of prednisolone at 0.6 mg/kg/day. Immunomodulators and rituximab should be tried in refractory cases [124].

#### CQ-IV-4) How should the glucocorticoid dose be tapered?

- After 2–4 weeks of the initial dose, glucocorticoid should be tapered by 5 mg every 1–2 weeks based on changes in clinical manifestations, biochemical blood tests, and repeated imaging findings. The dose is tapered to a maintenance dose (recommended more than 5 mg/day) over a period of 2–3 months (Level of recommendation: B).

 < Description > 

Radiological improvement appears 1 to 2 weeks after the initiation of glucocorticoid therapy. A poor response to glucocorticoid therapy should flag the possibility of pancreatic cancer and the need for re-evaluation of the diagnosis [121].

#### CQ-IV-5) Is glucocorticoid maintenance therapy necessary?

- Glucocorticoid maintenance  therapy is effective to prevent relapses of AIP, and administration of oral prednisolone should be maintained at doses of at least 5 mg/day (Level of recommendation: B).
- For the application of  glucocorticoid maintenance therapy, it is important to determine the disease activity based on imaging findings, serum IgG4 levels, and the presence or absence of extra-pancreatic lesions (Level of recommendation: B).

 < Description > 

Glucocorticoid maintenance therapy is common in Japan and South Korea, but not in Western countries [121, 125]. Efficacy of long-term glucocorticoid maintenance therapy in the prevention of relapses with oral prednisolone at doses of at least 5 mg/day has been shown in a randomized controlled trial from Japan [126] as well as a systematic review/meta-analysis of 36 studies [127].

The international consensus recommends glucocorticoid maintenance therapy in cases presenting diffuse enlargement of the pancreas, delayed radiological remission or persistently high serum IgG4 after treatment, or more than two extra-pancreatic lesions or association with proximal IgG4-sclerosing cholangitis before treatment [128].

#### CQ-IV-6) When should glucocorticoid therapy be discontinued?

- Glucocorticoid maintenance therapy following complete remission should be for around 3 years (Level of recommendation: A).
- Continuous follow-ups are recommended even after 3 years (Level of recommendation: B).
- Continuation after 3 years of maintenance therapy should be determined based on activity, and care should be taken towards adverse events from glucocorticoids (Level of recommendation: B).

 < Description > 

Long-term administration with a maintenance dose of around 5 mg/day for 3 years is recommended for relapse prevention based on the results of multiple retrospective trials [125] and randomized controlled trials [126]. Meanwhile, there is a risk of relapse even after the discontinuation of maintenance therapy [129, 130]. Difficulty with the discontinuation of glucocorticoids should be carefully considered for each case, while paying sufficient attention to the necessity of continued treatment and the occurrence of adverse events from glucocorticoids.

#### CQ-IV-7) Is early detection of relapse possible?

- Regular follow-up using serum biochemical tests, serum IgG4 levels, and imaging tests including those for extra-pancreatic lesions would enable the early detection of relapse (Level of recommendation: B).
- Relapse prediction includes discontinuing glucocorticoid therapy within a short time period, high serum IgG4 levels at the time of diagnosis, persistent high serum IgG4 levels following glucocorticoid therapy, diffuse pancreatic enlargement, bile duct lesions, and multiple extra-pancreatic lesions (Level of recommendation: B).

 < Description > 

Regular follow-ups based on serum biochemical tests, serum IgG4 levels, and imaging tests are effective for the early detection of relapse. Regular inspections with consideration for relapse risk are recommended when there are multiple relapse predictors [122, 125, 130].

#### CQ-IV-8) How should relapsed patients be treated?

- The re-administration of glucocorticoid therapy or higher doses of glucocorticoids is recommended (Level of recommendation: A)
- The combined use of glucocorticoids and immunosuppressants or rituximab is performed in the West for steroid-resistant or dependent patients. However, this treatment is not covered by public medical insurance in Japan, so the standards of the Clinical Research Act must be observed (Recommendation: None).

 < Description > 

The combined use of glucocorticoids and immunosuppressants or rituximab is effective for steroid-resistant or dependent patients [124, 131]. However, easy implementations are not permitted due to the reactivation risk of serious adverse events (e.g., serious infectious diseases and infusion reaction) and hepatitis B virus.

#### CQ-IV-9) Do pancreatic exocrine and endocrine functions improve after glucocorticoid therapy in AIP patients?

- Pancreatic exocrine and endocrine functions improve after glucocorticoid therapy in some AIP patients. The improvement rate is high in diabetes mellitus that develops with AIP simultaneously (Level of recommendation: B).

 < Description > 

Glucocorticoid therapy has been reported to improve pancreatic exocrine and endocrine function [27] in 38% [28] to 50% [29] and 25% [28] to 45% [29] of AIP patients, respectively. Additionally, it is reported that glucocorticoids improved control in about half of the cases of diabetes mellitus associated with AIP, and the improvement rate was high in diabetes mellitus that develops with AIP simultaneously [132]. Diabetes mellitus control was shown to worsen in 75% of AIP patients with diabetes mellitus before AIP onset [29]. AIP may transform into chronic pancreatitis and pancreatic atrophy occurs after glucocorticoid therapy [121].

#### CQ-IV-10) Is the prognosis of AIP favorable?

- AIP can be expected to have a good short-term outcome with glucocorticoid therapy (Level of recommendation: A).
- The long-term treatment effect and functional outcome of AIP may be less clear. Some AIP patients have a relapse during or after the glucocorticoid therapy. The functional outcome (e.g., exocrine and endocrine function) is not always good.

 < Description > 

According to a meta-analysis, the relapse rate after steroid treatment was 33% with an average observation period of 41 months [127]. It has been reported that some patients develop chronic pancreatitis and pancreatic dysfunction due to repeated relapse [29, 122].

#### CQ-IV-11) Is AIP a risk factor for pancreatic cancer?

- Some studies have reported that pancreatic cancer occurred with AIP, but its causality remains unknown and scientific evidence is lacking.

 < Description > 

The incidence rate of pancreatic cancer in patients with AIP is 0–4.8%. Most cases of pancreatic cancer are identified more than 1 year after the diagnosis of AIP [124, 133, 134], but some at the same time of diagnosis of AIP [135], which suggests that AIP might be a pre-cancerous condition or might sometimes arise from co-existing cancers as a paraneoplastic syndrome [136].
